# Esophageal granular cell tumor and eosinophils: a multicenter experience

**DOI:** 10.1186/s13000-021-01113-3

**Published:** 2021-06-08

**Authors:** Deepti Reddi, Christropher Chandler, Diana Cardona, Michael Schild, Maria Westerhoff, Emily McMullen, Yutaka Tomizawa, Lani Clinton, Paul E. Swanson

**Affiliations:** 1 Department of Laboratory Medicine and Pathology, University of Washington Medical Center, Box 356100, 1959 NE Pacific St, Seattle, WA 98195 USA; 2 Department of Pathology, Duke University Medical Center, Durham, NC 27710 USA; 3 Department of Pathology, University of Michigan, Ann Arbor, MI 48109-2800 USA; 4 Department of Medicine, Division of Gastroenterology, University of Washington, Seattle, WA 98195-6420 USA

**Keywords:** Granular cell tumor, Eosinophilic esophagitis, Goblet cell metaplasia

## Abstract

**Background:**

Esophageal granular cell tumor (eGCT) is rare, and the recent literature suggests a link between eosinophilic esophagitis (EoE) and eGCT. The aim of our study was to determine if EoE or other disorders associated with eosinophilia are consistently associated with eGCT.

**Methods:**

We retrospectively searched pathology databases of three academic institutions from 1999 to 2018 for eGCTs. The archived slides and medical records were reviewed.

**Results:**

From 294,855 esophagogastroduodenoscopy procedures, 45 patients (17 males and 28 females) with eGCTs were identified. The patients (30–73 years in age, median 50) had eGCT 0.2–2.0 cm in size (average 0.71). Thirteen had a history of gastroesophageal reflux disease, 5 had Barrett esophagus/goblet cell metaplasia and 1 had EoE. Thirty-four eGCTs had intralesional eosinophils (14 with peak > 10 eosinophils/400x hpf); of these, 21 also had eosinophils in lamina propria (9 with peak > 10 eosinophils/hpf). eGCT with atypical features (including nuclear enlargement and prominent nucleoli) were more likely to have increased eosinophils in non-epithelial compartments than those without atypia. Pleomorphism and spindled cells were seen in 3 eGCT cases (mean peak intralesional eosinophils: 43 per hpf); 2 of these had goblet cell metaplasia. We found no association between EoE and eGCT, *p* = 0.5966, (95% C.I. 0.0276, 6.5389, Fisher’s exact test). Instead, most patients had gastroesophageal reflux disease or Barrett esophagus.

**Conclusion:**

Eosinophilia, common in eGCT and adjacent stroma, likely drives atypical/reactive histologic features, but a pathogenic relationship between eosinophil rich inflammatory conditions and eGCT has not yet been established.

## Background

Granular cell tumor (GCT) was first described in the tongue by Abrikossoff in 1926. Other names for this entity are granular cell myoblastoma and Abrikossoff’s tumor [1, 2]. Although the lesion is most commonly seen in the skin, soft tissue and tongue, 8–11% of cases occur in the gastrointestinal tract [1–5]. In 1931, Abrikossoff first described GCT in the esophagus, which we now know is the most common site of involvement within the gastrointestinal tract, primarily the distal segment of the esophagus [3, 4].

In the esophagus, eosinophilic esophagitis (EoE) is a clinicopathologic disease that has been more consistently recognized, with increasing prevalence, since the initial publication of consensus recommendations in 2007 [6]. EoE is a chronic, immune/antigen-mediated disease with eosinophil-predominant inflammation that leads to esophageal dysfunction [7]. GCTs have been previously linked to sites of injury and inflammation, leading some to postulate that these lesions might be reactive in nature [8]. There have been several reported cases of concomitant esophageal granular cell tumor (eGCT) and EoE cases in both adults and pediatric patients [9–11]. More recently, two separate single center case series proposed an association between the two entities [12, 13]. In order to further study this possible link, we conducted a multi-center study including three separate academic center institutions from different regions of the United States.

## Methods

Pathology databases from University of Washington Medical Center (UWMC), Duke University Medical Center (DUMC) and University of Michigan Medical Center (UMMC) were retrospectively searched for cases of eGCT from January 1999 to January 2018. Patient medical records were reviewed for demographic information, diagnoses, presenting clinical symptoms, endoscopic findings and follow-up. A total of 108,244 EGD procedures were performed at UWMC from January 1999 to January 2018, and 1704 patients had diagnosis of EoE by ICD9 and ICD10 codes from 2008 to 2018. Endoscopy procedure data were available from DUMC from January 2006 to January 2018, where 77,295 esophagogastroduodenoscopy (EGD) procedures were performed, and 1481 patients had diagnosis of EoE by ICD9 and ICD10 codes. At UMMC, there were 64,316 EGD procedures and 2692 cases of EoE by ICD9 and ICD10 codes from June 2012 to May 2020.

Cases of eGCT were retrieved from the pathology archives of the three participating institutions and blindly reviewed by experienced GI pathologists practicing at each site. In addition to confirming the pathologic diagnosis, the pathologists also recorded typical histologic features including necrosis, spindling, nuclear pleomorphism, increased mitotic activity (≥2 mitosis/ 400x), increased nuclear size (at 100x), and large nucleoli (at 100x) for each case of eGCT [12, 14]. The lesional tissue, surrounding stroma and the overlying epithelium were evaluated for eosinophilia and the peak eosinophil count in each compartment was documented at 400x magnification (hpf). The statistical significance of observed versus expected rates of simultaneous eGCT and EoE was analyzed using Fischer’s exact test based on the null hypothesis of no association with a two-tailed *p*-value of < 0.05 considered statistically significant (calculated using STATA version 14, STATA Corp LP, TX).

## Results

We pooled data from 294,855 EGD procedures at three academic medical centers and identified 45 patients with eGCT (Table 1).

**Table 1:** Clinical and histologic findings of 45 patients with esophageal granular cell tumor

Patient no.	Age (yr)	Sex	Clinical symptom(s)	eGCT atypical feature(s)	Peak Eos/HPF in GCT	Peak Eos/HPF in lamina propria	Peak Eos/HPF in overlying epithelium
1	50	F	GERD	None	2	9	2
2	53	F	GERD	Mild increased nuclear size	4	10	5
3	58	F	GERD	None	0	0	0
4	53	M	GERD	None	49	43	2
5	41	F	Reflux	Increased nuclear size, nucleoli, pleomorphic	20	1	1
6	50	M	Reflux	Increased nuclear size, nucleoli	26	15	0
7	40	M	Heartburn, anemia, vomiting	None	0	2	0
8	49	F	Heartburn, Dysphagia	Increased nuclear size, nucleoli	0	NA	0
9	52	M	GERD	Increased nuclear size, nucleoli	33	39	0
10	48	F	Reflux and dyspepsia	Increased nuclear size, nucleoli, pleomorphic	0	4	0
11	58	F	GERD and pain	None	0	1	1
12	45	M	Heartburn and dysphagia	None	0	NA	0
13	33	M	GERD, diarrhea, weight loss	Increased nuclear size	1	NA	0
14	61	M	Barrett and duodenal adenoma	Increased nuclear size^a^	0	0	0
15	45	M	Barrett	None	2	6	1
16	50	M	Barrett	Increased nuclear size	13	8	60
17	66	M	Long segment Barrett	Increased nuclear size, nucleoli, pleomorphic, spindling^a^	6	5	NA
18	48	F	Atypical chest pain	Increased nuclear size, nucleoli, pleomorphic, spindling^a^	112	18	0
19	33	F	EoE and dysphagia	Increased nuclear size	2	1	82
20	52	M	Varices screen	Mild spindling	8	0	0
21	68	F	Nausea	None	8	0	1
22	53	F	Gastric bypass	Increased nuclear size, spindling	24	0	1
23	50	F	Dysphagia	None	7	3	1
24	56	M	Dysphagia and weight loss	None	1	NA	0
25	57	F	Hematemesis	Increased nuclear size, spindling	14	3	0
26	39	F	Odynophagia	None	1	NA	1
27	73	F	Anemia	None	0	NA	0
28	42	F	Gastric bypass	None	4	5	1
29	52	M	Esophageal SCC	None	5	NA	49
30	57	F	Pain and anemia	Increased nuclear size, nucleoli, pleomorphic	37	30	4
31	44	F	chronic pancreatitis	None	11	NA	1
32	48	F	Dysphagia	None	35	12	5
33	30	M	Melena	None	0	0	1
34	55	F	NA	None	4	8	0
35	54	F	Esophageal stricture	None	29	NA	7
36	55	F	Dysphagia and lichen planus	None	1	NA	0
37	49	F	Anemia	None	0	NA	0
38	54	M	Anemia	Increased nuclear size, nucleoli	11	29	NA
39	48	M	Dysphagia, prior GCT history	Increased nuclear size, nucleoli, pleomorphic, spindling	12	51	0
40	47	F	Dyspepsia and anemia	None	0	0	0
41	51	F	Dysphagia	Increased nuclear size	3	NA	0
42	47	F	Diarrhea, n/v, pain	Increased nuclear size, nucleoli, pleomorphic	6	3	0
43	42	F	Prior GCT history	None	3	8	1
44	41	F	Prior GCT history	Increased nuclear size, nucleoli	2	1	0
45	55	M	Dysphagia, dyspepsia and vomiting	None	7	0	7

*Abbreviations*: *F* Female, *M* Male, *NA* Not available, *n/v* Nausea and vomiting, *Eos* Eosinophils

^a^Goblet cell metaplasia

### Clinical findings

All 45 patients were adults; 17 were males and 28 were females (male to female ratio of 1:1.65). Ages ranged from 30 to 73 years, with a median of 50. The most common presenting symptoms were dysphagia, gastroesophageal reflux disease and abdominal pain. Other diseases that were reported clinically or by histology were gastroesophageal reflux disease (13 patients), Barrett esophagus/goblet cell metaplasia (5 patients) and EoE (1 patient). There were three patients with prior history of eGCT by biopsy or fine needle aspirate diagnosis who later underwent endoscopic resection. The majority of patients had their lesion removed by endoscopic mucosal resection. Patient follow-up ranged from 0 to 216 months, with an average of 45. No patient had evidence of metastatic eGCT.

### Endoscopic findings

The size of the eGCTs ranged from 0.2–2.0 cm, with an average of 0.71. The most common site was the distal segment of the esophagus and most patients presented with a single nodule. There were two patients with multiple nodules, one of whom had multifocal disease involving all segments of the esophagus.

### Histologic findings

Specimens submitted for histopathologic evaluation were derived from either endoscopic biopsies (42%) or endoscopic mucosal resections with or without additional sampling of the esophagus (58%). Two patients had eGCT as an incidental finding during microscopic evaluation. One had eGCT in the proximal segment with a concomitant history of EoE with rings; the other underwent esophagogastrectomy for squamous cell carcinoma status post neoadjuvant therapy and was incidentally found to have eGCT in the distal segment.

**Fig. 1:**
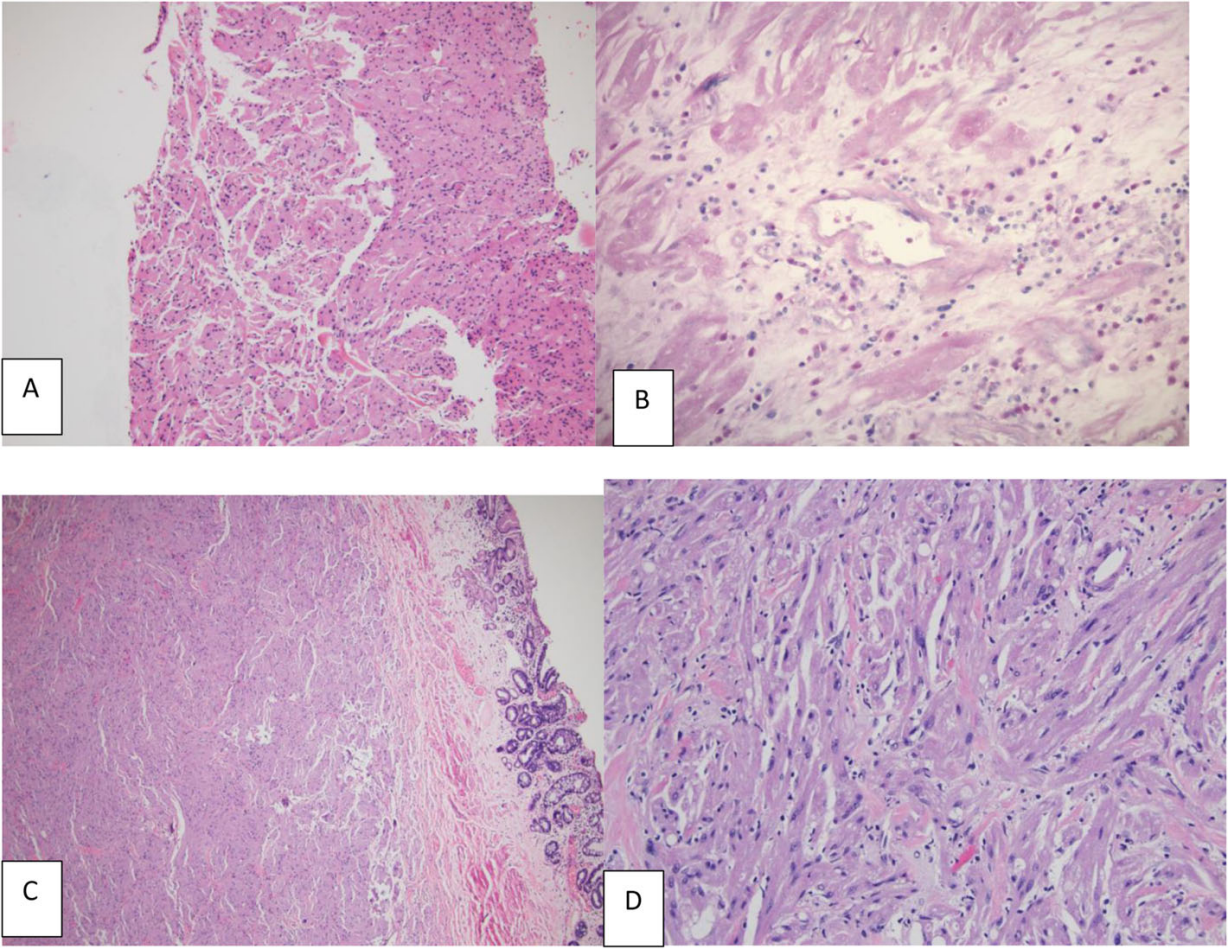
**A**, Incidental eGCT in an EoE patient with minimal atypia and lack of intralesional eosinophils (hematoxylin and eosin stain [H&E] at 100x). **B**, Atypical eGCT with significant intralesional eosinophilia (Geimsa histochemical stain at 200x). **C**, **D**, Biopsy from a Barrett esophagus and eGCT showing overlying epithelium with goblet cell metaplasia (H&E at 40x) and atypical GCT with areas of spindling, pleomorphism, increased nuclear size and prominent nucleoli (H&E at 200x)

In the single case of EoE, the accompanying eGCT had minimal cytologic atypia and few intralesional eosinophils (Fig. 1A). Thirty-four eGCTs had intralesional eosinophils (14 cases with peak > 10 eosinophils/400x hpf); of these, 21 also had eosinophils in lamina propria (9 cases with peak > 10 eosinophils/hpf).

Twenty-one cases of eGCT had atypical features; fourteen of these had ≤2 atypical features (increased nuclear size and presence of prominent nucleoli). In these cases, the mean peak intralesional eosinophil count was up to 10 eosinophils per 400x hpf. The remaining seven cases with atypia had additional atypical features (spindling and nuclear pleomorphism), and the peak intralesional eosinophils for these cases was up to 112 eosinophils per 400x hpf (Fig. 1B).

Of the 5 cases with Barrett esophagus/goblet cell metaplasia (Fig. 1C), two had 4 atypical features in the accompanying eGCT with areas of spindling, pleomorphism, increased nuclear size and prominent nucleoli (Fig. 1D). No cases of eGCT had necrosis or increased mitosis. In addition, there was no association between eGCT with atypical features and the patient’s age.

Considering all EGD procedures performed at the three institutions with a diagnosis of EoE and eGCT, we found no association between EoE and eGCT, [*p* = 0.5966, (95% C.I. 0.0276, 6.5389, Fisher’s exact test)]. Considering the single case of eGCT associated with EoE from the UWMC cohort in isolation, there was still no association between EoE and eGCT [*p* = 0.3058, (95% C.I. 0.0688, 17.6042, Fisher’s exact test)].

## Discussion

EoE is an antigen-driven allergic condition with both genetic and environmental contributions [15, 16]. Although EoE occurs in most racial and ethnic groups, there is a predominance in non-Hispanic whites [7]. EoE is more common in patients from rural regions and cold climate zones; it is inversely associated with *Helicobacter pylori* infection both in adult and pediatric studies [16]. In our study, we attempted to control for inherent differences in patient population and geographical region by collating data from multiple tertiary care centers located in different regions of the United States.

Recent studies have reported the co-occurence of eGCT and significant esophageal intraepithelial eosinophilia, most frequently in the pediatric population [9, 11, 12]. In our multicenter retrospective study of eGCT from 294,855 EGD procedures, 40% of cases had gastroesophageal reflux disease or Barrett esophagus, but only one case in our series of 45 adults had concomitant EoE and eGCT (2%). The apparent rate of concomitant EoE and eGCT in our adult cohort is considerably less than that observed in prior studies, both of which included pediatric patients. Riffle and colleagues identified 18 patients with eGCT from among > 30,000 esophageal cases in their study, 33% of whom (*n* = 6) also had EoE. Four of these patients were adults and two were adolescents [12]. Similarly, Nojkov and co-authors reported 16 cases of eGCT their series of 167,434 EGD procedures, 31% of which (*n* = 5) were associated with EoE. Four of these were identified in adults, while 1 was diagnosed in an adolescent [13]. Interesting, although the absence of pediatric patients in our multi-institutional cohort likely affected our observed rate of concomitant EoE and eGCT, this does not completely account for the differences between our study and the prior retrospective studies. A larger multicenter prospective study may be warranted to further investigate the reported association and to determine the underlying pathophysiology of EoE and eGCT.

In addition to the single case of EoE in our eGCT group, there were two cases with significant intraepithelial eosinophilic infiltrates. One was a resection specimen from a patient with squamous cell carcinoma status post neoadjuvant therapy with complete response. The other case had significant eosinophilic infiltrates in biopsies from the distal esophagus, suggesting a closer relationship to severe reflux esophagitis. Reflux disease or heart burn was the presenting symptom in 28% of our patients. The eGCTs found in these patients were in the distal segment of the esophagus (66%), ranging in size from 0.3 to 2.0 cm, in greatest dimension. These findings raise the possibility that the eGCT, by potentially impairing function of the gastroesophageal junction, may have contributed to the onset or severity of reflux esophagitis in these patients (a case report of an esophageal leiomyoma causing reflux esophagitis emphasizes this point [17]). It is tempting, with these observations in mind, to suggest the inclusion of mass lesions including eGCT in the differential diagnosis of new onset reflux esophagitis.

There are reports of concomitant presentation of eGCT and other neoplasms, both benign and malignant, including leiomyoma, squamous cell carcinoma and intramucosal adenocarcinoma in the setting of Barrett esophagus [18–20]. The latter illuminates one of the more interesting and novel findings of our study: the presence of eGCT in patients with an established diagnosis of Barrett esophagus or histologic evidence of goblet cell metaplasia. It is also of interest that cases of eGCT with background goblet cell metaplasia showed more atypical features, along with significantly increased intratumoral eosinophils.

Interestingly, neither Nojkov et al. nor Riffle et al. identified eGCT patients with Barrett esophagus/goblet cell metaplasia. Nonetheless, the latter report included observations similar to those in our study, including a high number of eGCTs (67%) with increased intratumoral eosinophilia and a disproportionately high number of eosinophils in eGCT with atypical features [12]. In our study, 76% of eGCT cases had increased intralesional eosinophils and 47% of the cases had eosinophils in the lamina propria of the overlying esophageal mucosa. Given the reproducibility of these findings, we postulate that eosinophilia within the eGCT and adjacent stroma may be driving the observed atypical/reactive histologic features. Although eGCT is commonly considered a benign entity; there are rare reports of cases that presented with lung and liver metastases. There is also a single report of eGCT secreting a tumor marker (carbohydrate antigen 19–9) [21–24]. Notably, we had no cases of metastatic eGCT in our patient cohort.

Our retrospective study of eGCT is the largest to date, drawing on patient populations from three tertiary care medical centers located in different geographic regions of the country. Even so, our investigation has limitations. Most significantly, our patient cohort consists of only adults and thus differs from the prior published studies. We had two eGCT that were diagnosed incidentally by histology because of procedures for other conditions. All other cases were initially diagnosed in standard esophageal biopsies, a sample type that is typically limited in usual practice, containing only the esophageal epithelium that is immediately superficial to the lesion. With the lack of submucosa in most biopsy specimens, our sample set likely underestimates the prevalence of eGCT in these populations. Also of note, nine patients in our cohort were diagnosed within the 2001–2007 year period, before consensus guidelines for EoE was established [6, 7]. If EoE was widely recognized as a disease entity earlier, more diagnostic and follow up material would have been available for histologic evaluation and we may have identified additional cases of eGCT in the setting of esophageal eosinophilia.

## Conclusion

Most patients with eGCT in our cohort had gastroesophageal reflux disease (29%) or an established diagnosis of Barrett esophagus (11%). These patients have frequent follow up with higher rate of endoscopy; hence, the finding of eGCT may be coincidental. Importantly, we found no association between EoE and eGCT. We hypothesize that eosinophilia within the lamina propria of esophageal mucosa likely drives atypical/reactive histologic features within eGCT; however, a pathogenetic relationship between eosinophil mediated inflammatory conditions (including EoE) and eGCT is not yet established.

## Data Availability

All data generated or analyzed during this study are included in this published article.

## Abbreviations

GCT: Granular cell tumor
EoE: Eosinophilic esophagitis
eGCT: Esophageal granular cell tumor
UWMC: University of Washington Medical Center
DUMC: Duke University Medical Center
UMMC: University of Michigan Medical Center
EGD: Esophagogastroduodenoscopy
